# Exercise Therapy for Management of Type 2 Diabetes Mellitus: Superior Efficacy of Activity Monitors over Pedometers

**DOI:** 10.1155/2016/5043964

**Published:** 2016-09-28

**Authors:** Masaaki Miyauchi, Masao Toyoda, Noriko Kaneyama, Han Miyatake, Eitaro Tanaka, Moritsugu Kimura, Tomoya Umezono, Masafumi Fukagawa

**Affiliations:** Division of Nephrology, Endocrinology and Metabolism, Department of Internal Medicine, Tokai University School of Medicine, Kanagawa, Japan

## Abstract

We compared the efficacy of activity monitor (which displays exercise intensity and number of steps) versus that of pedometer in exercise therapy for patients with type 2 diabetes. The study subjects were divided into the activity monitor group (*n* = 92) and pedometer group (*n* = 95). The primary goal was improvement in hemoglobin A1c (HbA1c). The exercise target was set at 8,000 steps/day and 20 minutes of moderate-intensity exercise (≥3.5 metabolic equivalents). The activity monitor is equipped with a triple-axis accelerometer sensor capable of measuring medium-intensity walking duration, number of steps, walking distance, calorie consumption, and total calorie consumption. The pedometer counts the number of steps. Blood samples for laboratory tests were obtained during the visits. The first examination was conducted at the start of the study and repeated at 2 and 6 months. A significant difference in the decrease in HbA1c level was observed between the two groups at 2 months. The results suggest that the use of activity level monitor that displays information on exercise intensity, in addition to the number of steps, is useful in exercise therapy as it enhances the concept of exercise therapy and promotes lowering of HbA1c in diabetic patients.

## 1. Introduction

Diet and exercise therapy form the basis of treatment of type 2 diabetes mellitus (T2DM). These two approaches are well known to improve blood glucose control [1]. Exercise therapy has also been reported to be effective in improving blood glucose control and quality of life (QOL) [2, 3]. However, reduction of fat and improvement in insulin resistance are limited with diet modification alone [4].

As such, even while the effects of exercise therapy are well known, in reality, even when diet therapy is followed nearly by all patients, the percentage of patients who adhere to exercise therapy is only about 40% [5]. The reasons for this low rate are that exercise therapy is not easy to implement in patients with no physical training background, time restrictions, and inability to maintain motivation. Another reason is that the actual techniques and goals of exercise therapy are often difficult to understand by both the patient and the instructor.

Target indicators for exercise therapy include both the number of steps and strength [5], and moderate-intensity training is considered particularly effective. While 3–6 metabolic equivalents (METs) are proposed for exercise therapy of moderate intensity [5, 6], in actuality, checking the intensity level during training sessions is often difficult. For effective exercise therapy, the activity level should be appropriately increased by monitoring and being aware of the exercise intensity. Development of a simple and useful tool toward this end would help improve the outcome of treatment of T2DM. Using a conventional device that measures the number of steps (pedometer) and another device that measures the number of steps and exercise intensity and amount (activity monitor), the present study was designed to evaluate the effects of exercise therapy with awareness of training intensity with regard to improvement in blood glucose control.

## 2. Materials and Methods

### 2.1. Patients and Methods

The subjects were 200 adult patients with T2DM who visited our division at Tokai University Hospital between March and April 2012 and were judged by their physicians as fit to receive exercise therapy. In addition, at the beginning of the study, the methods and purpose of the research and the voluntary nature of cooperation were explained verbally and in writing, and written agreement was obtained from all patients. This study was registered as a clinical trial (UMIN000018694), with the inspection and approval of the institutional review board for clinical research of Tokai University Hospital.

The number of steps and amount of physical activity were recorded digitally using an activity monitor (model MT-KT01, Terumo, Tokyo, Japan) with a triaxial speed sensor that measures the number of steps and the time spent walking at a moderate-intensity level. Another device, a modified MT-KT01, was used as a pedometer to count the number of steps during walking/exercise.

**Figure 1:**
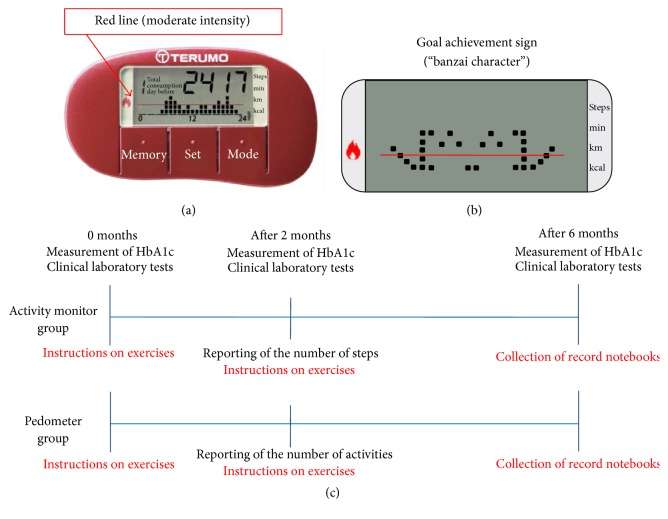
(a) On the activity monitor, for exercise of moderate intensity of 3 METs or higher, the intensity is displayed when the indicator exceeds the red line. (b) If the daily goal of moderate-intensity exercise of 20 minutes or longer and at least 8,000 steps is set and achieved, the user is notified that the goal has been achieved through a sign on the screen (a “banzai character”). (c) Study design.

After randomly assigning 100 subjects each to the activity monitor group and the pedometer group, a target was set for the pedometer group, which was walking exercise of moderate intensity (3 METs or higher) for least 20 minutes a day and 8,000 steps. The same target of at least 3 METs (at or above the achievement line indicator in the activity monitor; Figure 1(a)) for a minimum of 20 minutes a day of exercise and 8,000 steps was also set for the activity monitor group. Both the pedometer and activity monitor were hung from a strap around the user's neck during waking hours.

The achievement of the target exercise was signaled by a display of the goal achievement sign (Figure 1(b)) on the activity monitor. The patients were asked to manually record the data of their activity monitor and pedometer in record sheets, which were collected during the outpatient visits. Clinical data measurements, including HbA1c level, were performed during the outpatient visits, with evaluation of the number of steps and target achievement ratio in the second month and final evaluation in the sixth month (Figure 1(c)).

**Figure 2:**
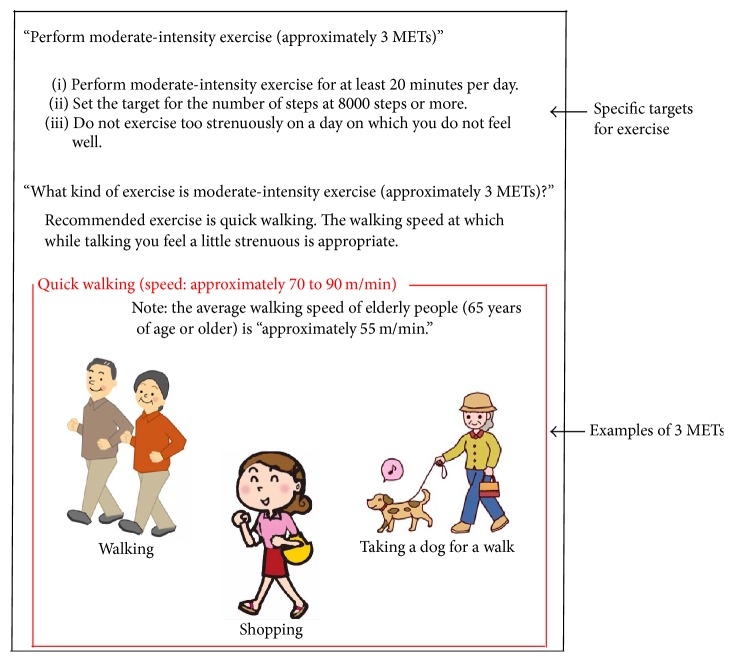
The explanatory pamphlet for exercise therapy. Examples of moderate-intensity exercises for the patients who participated in the study of both the activity monitor and the pedometer groups. The exercise goal of at least 20 minutes and 8,000 steps a day was based on the pamphlet.

Instructions were provided regarding the exercise on a pamphlet handed to each patient (Figure 2) at the beginning of the study. At 2 months after the start of training, the subjects were asked to report the number of steps and amount of exercise, as well as whether they had achieved the set targets. Those who self-reported that they had achieved the goals were instructed to continue, while those who had not done so were again provided information described in the pamphlet without any new intervention.

### 2.2. Statistical Analysis

**Figure 3:**
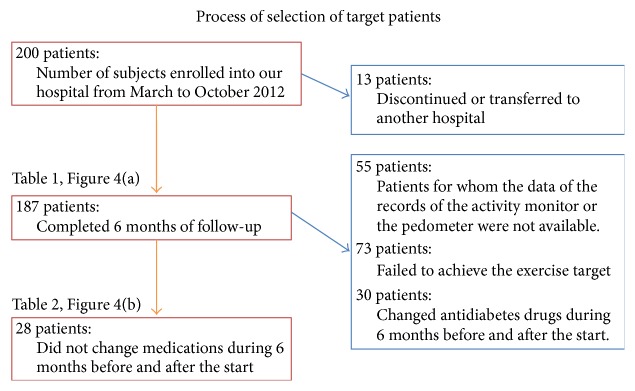
Patient selection process. After excluding those patients who cancelled, transferred to other hospitals, or dropped out, the data of 187 patients were subjected to analysis. After excluding patients with insufficient exercise therapy record data, unachieved exercise goals, and changes in medications in the 6 months before and after the start of the study period, data of 28 patients of each group were compared and studied.

The 187 patients who completed their 6-month follow-up were the subjects of the analysis (Figure 3). The pedometer and activity monitor data were compared, as well as changes in medications. Concerning continuation of exercise and achievement of targets, the analysis defined those with at least 80% of day count data and at least 80% of target exercise amounts as meeting the goals.

The HbA1c levels at the beginning of the study and at 2 and 6 months later were compared as main items by performing responsive examination. The Pearson chi-square test or Mann-Whitney *U* test was used for comparison of variables between the two groups, and the significance level was set at 5%. The statistical analysis software used was JMP Ver. 11.0.0 (SAS Institute Japan, Tokyo).

## 3. Results

### 3.1. Patients Background Characteristics and Changes in HbA1c Level

**Figure 4:**
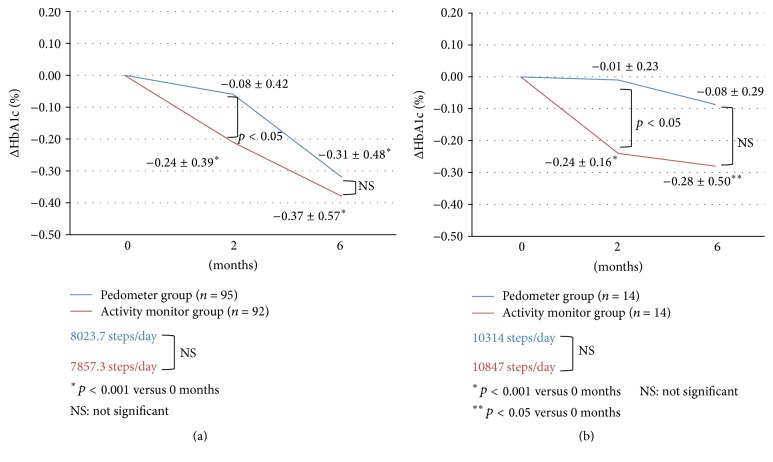
(a) Changes in HbA1c level after daily walking exercise for 2 and 6 months in the pedometer and activity monitor groups and all 187 patients. (b) Changes in HbA1c level after daily walking exercise for 2 and 6 months in 28 patients of the pedometer and activity monitor groups who achieved their goals and recorded no changes in medications throughout the study.

The clinical background data of the 187 patients who completed the 6-month follow-up are summarized in Table 1, and the delta changes in HbA1c level during the study are shown in Figure 4(a).

Based on the background characteristics of all the 187 patients, no clear difference was found between the two groups other than a significant preponderance of men in the activity monitor group and the value of uric acid in the blood being significantly low in the pedometer group (Table 1).

With regard to changes in HbA1c level, significant reductions in HbA1c level at 2 and 6 months after the start of the study were observed in the activity monitor group, compared to that before the start of study. Comparison of data of the two device groups showed a significant difference in the level of reduction in HbA1c level at 2 months between the pedometer and activity monitor groups (pedometer group: 0.08 ± 0.42%, activity monitor group: 0.24 ± 0.39%). However, no significant difference was observed between the two groups at 6 months.

**Figure 5:**
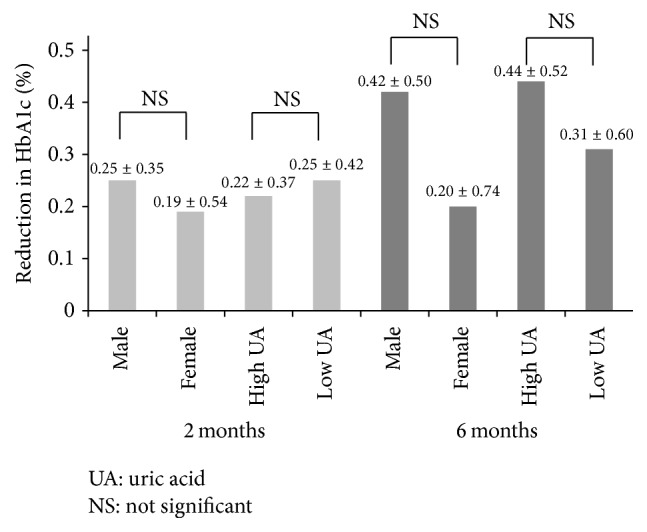
Comparison of the levels of reduction in HbA1c at 2 and 6 months according to sex and serum uric acid.

Changes in HbA1c at 2 and 6 months were also compared according to sex and uric acid level. There was no significant difference in HbA1c between males and females (Figure 5). After dividing the patients into those with high and low uric acid levels, using the median uric acid level as the cutoff value, we found no difference between the two groups (Figure 5).

Next, we excluded the data of 55 subjects with physical activity data less than 80% of those recorded at 6 months and 73 subjects with physical activity data less than 80% of the exercise target achievement rate. Thus, 59 patients continued the exercise therapy for 6 months, including 36 of the activity monitor group and 23 of the pedometer group. The continuation rate was 37.9% and 25.0%, respectively. The exercise therapy continuation rate of the activity monitor group was significantly better than that of the pedometer group (*p* = 0.0282).

Since no limitation was imposed in the present study on the use and changes in medications, the effects of the drugs were excluded. For meaningful analysis, however, we selected those patients in whom no changes in medications were made 6 months before and after the start of the study (i.e., for 1 year) and analyzed their data for the effects of exercise therapy only. The results showed that changes in HbA1c level purely due to exercise were noted in 14 subjects from each group (Figure 4(b)).

Significant reductions in HbA1c level from the time before to the time after the start of the study were observed in the activity monitor group but not in the pedometer group both at 2 and at 6 months. The decrease in HbA1c level at 2 months was significantly larger in the activity monitor group (0.24 ± 0.16%) compared with the pedometer group (0.01 ± 0.23%). A similar trend was noted at 6 months, though the difference was not significant.

## 4. Discussion

The US guidelines recommend 150 minutes of exercise per week as appropriate exercise therapy. However, a recent study indicated that 90 minutes per month of low to moderate exercise is beneficial in Asians [7]. Thus, fast walking, which can be easily achieved on a daily basis, is considered moderately intense exercise in Asia. For this reason, this study was conducted by selecting walking exercise as the exercise therapy, which is considered the easiest to perform. This study was planned with a hypothesis that, as a resolution to the issue of “I do not have time to exercise,” changing the daily activity itself to a level of moderate intensity is a sufficient exercise therapy, even if one just cannot take time to exercise, and that activity monitors have better results than pedometers as a measure of exercise efficacy.

The target activity was set in the present study at 3 METs, although moderately intensive exercise is effective in exercise therapy in T2DM patients [8]. Unconditionally speaking, even for moderate-intensity exercise, the exercise burden should be adjusted according to age. For those patients aged ≥ 65 years (constituting the majority of our patients and representing the majority of patients with T2DM in Japan), the 3-MET level is considered of moderate intensity [6]. We therefore set 3 METs and higher as the target activity in this study.

Our results showed reduction of HbA1c level in both groups when the patients of both groups exercised while being aware of the 3-MET target. These results confirmed that exercise therapy, at least for 6 months, contributed to the improvement in HbA1c in patients with T2DM. Furthermore, patients who wore the activity monitor, which provided feedback about their exercise intensity, also showed reduction in HbA1c level, which was significantly better at 2 months compared with the pedometer group. This difference was thought to be due to the motivation to exercise at a moderate level, combined with feedback from checking exercise intensity, with resultant more beneficial effects in the activity monitor group than the pedometer group. Considered together, these results suggest that the use of activity monitor seems to enhance the reduction in HbA1c level.

As shown in Figure 4(b), in the study that excluded the effects of medications, the decrease in HbA1c level was interestingly larger in the activity monitor group than in the pedometer group at both 2 and 6 months. This finding suggests that using an activity monitor is important in exercise therapy, as it provides information about exercise intensity, and that such monitor is particularly effective in the simple exercise therapy of walking as part of daily activities.

Furthermore, the most noteworthy result of this study was the difficulty of continuing the exercise therapy in the 6-month study period. Thus, the percentage of patients who continued exercise therapy for 6 months at ≥80% of the exercise therapy was less than 40% in both groups: 37.9% of the activity monitor group and 25.0% of the pedometer group, indicating the difficulty in continuing exercise therapy itself. Based on these results, we emphasize the need for motivating T2DM patients to continue exercise.

With regard to the provision of instructions or guidance to the patients regarding exercise therapy, in addition to its usefulness in maintaining motivation through the ability to recognize exercise intensity levels, even in patients who received instructions through the pamphlet only, checking that the target of 3 METs has been achieved during walking is useful in learning the appropriate walking speed. Therefore, this is possible not only by a physician but also through instruction provided by other medical staff. This demonstrates that the mere act of presenting exercise goals to patients before using activity monitors and incorporating moderate to high-level movement into their daily activities seemed to contribute to the efficacy of exercise therapy.

The present study has certain limitations. The reduction in HbA1c level at 6 months coincided with the summer season (from the fourth month after the start of the study). People tend to stay less outside for reasons such as to avoid heatstroke. Due to potential reduced physical activity and the effects of increased intake of glucose-rich sports drinks, further studies of longer duration are needed, especially studies that take seasonal variations into consideration. Table 1 shows that the percentage of males was significantly higher in the activity monitor group than in the pedometer group. This could contribute to selection bias. For this reason, we compared the extent of reduction in HbA1c at 2 and 6 months between males and females and between patients with high and low uric acid (Figure 5). We also analyzed background characteristics and HbA1c changes in 28 patients who did not change their medications during the 6-month period (Table 2). Another limitation of the present study is that it compared only the number of steps but not other parameters that could be used to evaluate whether the use of activity monitors actually increased the total amount of physical activity (e.g., walking duration, walking distance, and total calorie expenditure) in the two groups. Unfortunately, such data could not be stored in the pedometer device.

The only available explanation for the continuation of exercise by patients who did not achieve the exercise goals was the second explanation of the pamphlet provided during the consultation in the second month. However, it is difficult to say that this is completely the same as instructions given about exercise therapy in daily clinical consultation. Further studies are needed to select the best follow-up regimen and its relationship with the achievement of exercise targets in patients with T2DM.

## 5. Conclusions

We have demonstrated in the present study the importance of exercise therapy for patients with T2DM. The results showed that awareness of the level of exercise intensity through the use of an activity monitor that provides information about exercise intensity, not a pedometer, improved HbA1c level in the initial period of exercise. The results suggest that the use of devices with functions that allow verification of goal achievement in concrete terms contributes to the continuation of exercise therapy among patients.

## Figures and Tables

**Table 1:** Clinical characteristics of the activity monitor group and pedometer group.

	Activity monitor group (*n* = 92)	Pedometer group (*n* = 95)	*p* value
Age (years)	62.7 ± 9.2	62 ± 10.6	0.97
Male	78.3%	56.8%	<0.005
Height (m)	1.63 ± 0.08	1.62 ± 0.09	0.12
Weight (kg)	72.6 ± 15.7	69.1 ± 15.4	0.11
BMI	27.2 ± 5.1	26.4 ± 5.4	0.21
HbA1c (%)	7.1 ± 1.1	7.0 ± 1.2	0.53
Systolic BP (mmHg)	122.8 ± 11.1	123.0 ± 10.9	0.98
Diastolic BP (mmHg)	70.9 ± 9.3	71.9 ± 9.5	0.79
UA (mg/dL)	6.0 ± 1.5	5.3 ± 1.3	<0.005
HDL cholesterol (mg/dL)	59.9 ± 17.2	60.9 ± 19.7	0.85
LDL cholesterol (mg/dL)	108.7 ± 24.9	112.0 ± 23.9	0.60
Triglycerides (mg/dL)	151.1 ± 103.8	138.3 ± 75.9	0.49

Values are mean ± SD.

BMI: body mass index; BP: blood pressure; UA: uric acid; HDL: high-density lipoprotein; LDL: low-density lipoprotein.

**Table 2:** Clinical background of patients in whom medications were not changed throughout the study.

	Activity monitor group (*n* = 14)	Pedometer group (*n* = 14)	*p* value
Age (years)	65.8 ± 6.7	62.4 ± 9.9	0.58
Male	78.6%	71.4%	0.66
Height (m)	1.61 ± 0.08	1.63 ± 0.09	0.46
Weight (kg)	62.4 ± 12.7	68.3 ± 9.5	0.08
BMI	24.1 ± 4.0	25.9 ± 4.3	0.24
HbA1c (%)	6.6 ± 0.6	6.4 ± 0.9	0.27
Systolic BP (mmHg)	121.1 ± 10.8	121.6 ± 6.8	0.89
Diastolic BP (mmHg)	67.8 ± 8.1	73.5 ± 9.0	0.09
UA (mg/dL)	5.5 ± 1.6	5.6 ± 1.6	0.80
HDL cholesterol (mg/dL)	66.6 ± 18.7	60.3 ± 11.6	0.72
LDL cholesterol (mg/dL)	111.4 ± 34.9	103 ± 10.8	0.09
Triglycerides (mg/dL)	107.6 ± 59.7	127.1 ± 90.8	0.68

Values are mean ± SD.

BMI: body mass index; BP: blood pressure; UA: uric acid; HDL: high-density lipoprotein; LDL: low-density lipoprotein.
